# EPR Studies of DOPA–Melanin Complexes with Netilmicin and Cu(II) at Temperatures in the Range of 105–300 K

**DOI:** 10.1007/s00723-012-0340-y

**Published:** 2012-05-13

**Authors:** Magdalena Zdybel, Barbara Pilawa, Ewa Buszman, Dorota Wrześniok, Ryszard Krzyminiewski, Zdzisław Kruczyński

**Affiliations:** 1 Division of Laboratory Medicine, Department of Biophysics, School of Pharmacy, Medical University of Silesia in Katowice, Jedności 8, 41-200 Sosnowiec, Poland; 2 Division of Laboratory Medicine, Department of Pharmaceutical Chemistry, School of Pharmacy, Medical University of Silesia in Katowice, Jagiellońska 4, 41-200 Sosnowiec, Poland; 3 Department of Medical Physics, Institute of Physics, Adam Mickiewicz University in Poznań, Umultowska 85, 61-614 Poznań, Poland

## Abstract

The application of electron paramagnetic resonance (EPR) spectroscopy in pharmacy of melanin complexes with netilmicin and Cu(II) was presented. The continuous microwave saturation of EPR spectra of DOPA–melanin and the complexes was performed. EPR spectra were measured on an X-band (9.3 GHz) spectrometer at temperatures in the range of 105–300 K. Paramagnetic copper ions decrease the intensity of the EPR lines of melanin’s free radicals. It was found that fast spin–lattice relaxation characterizes DOPA–melanin–Cu(II) complexes. Slow spin–lattice relaxation processes exist in melanin’s paramagnetic centers of DOPA–melanin and DOPA–melanin–netilmicin, [DOPA–melanin–netilmicin]–Cu(II), [DOPA–melanin–Cu(II)]–netilmicin complexes. Spin–lattice relaxation processes are faster at higher temperatures. The homogeneous broadening of EPR lines for melanin complexes was observed. The practical consequences of differences between paramagnetic properties of melanin complexes with netilmicin and the complexes with Cu(II) were discussed.

## Introduction

Melanins are paramagnetic biopolymers with the high content of *o*-semiquinone free radicals [1–5], which play an important role during the formation of the polymer complexes with drugs [6, 7]. Melanins bind a large number of drugs, such as gentamicin, kanamycin, netilmicin, dihydrostreptomycin, ciprofloxacin, lomefloxacin, norfloxacin and sparfloxacin [6–12]. It was shown that the free radical concentrations in melanin change after binding the drugs [6, 7, 11, 12]. The drug complexation by melanins causes their prolongated interactions in human organism during therapeutical application [13, 14]. These interactions depend on paramagnetic properties of the melanin polymer and they are modified by metal ions [11, 12].

In human body, melanin is found in skin, hair, eyes, inner ear, central nervous system and cardiac myocytes [15–17]. Melanin protects the pigmented tissues and the adjacent tissues through the absorption of pharmacologic agents, which are then slowly released in nontoxic concentrations. On the other hand, long-term medical treatment can lead to toxic concentrations of therapeutic substances in melanin and increased amounts of free radicals, which ultimately can lead to the tissue degeneration [13, 14].

Free radicals in natural and synthetic melanins were studied by electron paramagnetic resonance (EPR) spectroscopy [1, 3, 4, 6, 15, 17–21]. EPR spectra of *o*-semiquinone free radicals were obtained for melanin from hair [2, 16–18, 22], *Cladosporium cladosporioides* [19, 23], *Drosophila melanogaster* [20, 24], human substantia nigra [4, 25] and human eyes [15]. The lineshape of EPR spectra depends on the type of melanin existing in a biological system. For example, single EPR lines characteristic for eumelanin were measured for melanin biopolymer from human eyes [15] and black strain of *D. melanogaster* [20]. Pheomelanin with the complex spectrum with unresolved hyperfine structure was found in pigmented soil fungi *C. cladosporioides* [19, 23]. Neuromelanins reveal single or two-component EPR spectra [4, 26].

In addition to *o*-semiquinone free radicals with spin *S* = 1/2 [1, 4], biradicals with spin *S* = 1 were found in melanins [11, 12, 20]. The intensities (*I*) of EPR lines of melanin free radicals (*S* = 1/2) follow the Curie law (*I* = *C*/*T*). Biradicals (*S* = 1) do not follow the Curie law and another function describes the correlation between the intensity (*I*) and temperature (*T*).

Diamagnetic metal ions increase the intensity of melanin EPR lines [6, 12, 19] and paramagnetic metal ions decrease the free radical concentration in melanin samples [4, 6, 11, 12, 26–28]. Paramagnetism of DOPA–melanin complexes with netilmicin and Zn(II) and Cu(II) ions was described in Ref. [12]. Properties of the paramagnetic center system of melanin complexes with netilmicin, diamagnetic Zn(II) and paramagnetic Cu(II) metal ion complexes at room temperature were described by us earlier [6]. The aim of this work was to examine the effect of the measuring temperature on spin–lattice relaxation processes in melanin complexes with netilmicin and Cu(II).

This work expands the knowledge about paramagnetic properties of melanins. It is expected that paramagnetic centers of melanin play an important role during ultraviolet (UV) irradiation of organism. The free radicals formed during irradiation may interact with paramagnetic centers of melanin [8, 22, 29–33] and its complexes with drugs, so the knowledge about paramagnetic properties of melanin is very important from practical point of view. In this work paramagnetic properties of the exemplary melanin–antibiotic complexes were studied. Our results were compared with the known results obtained for melanin complexes with the others drugs [7, 10, 11].

## Materials and Methods

### Materials

DOPA–melanin, DOPA–melanin–netilmicin, DOPA–melanin–Cu(II), [DOPA–melanin–netilmicin]–Cu(II) and [DOPA–melanin–Cu(II)]–netilmicin complexes were examined. Synthetic DOPA–melanin was formed by oxidative polymerization of 3,4-dihydroxyphenylalanine (l-DOPA) in 0.07 M phosphate buffer at pH 8.0 according to Binns method [34]. DOPA–melanin—the model eumelanin was studied, because eumelanin is the main melanin polymer, which exist in human organism [1, 35].

Netilmicin is a popular aminoglycoside antibiotic [36]. It is a semisynthetic aminoglycoside analog of sisomicin [36, 37]. Netilmicin is characterized by the lowest toxicity of these aminoglycoside antibiotics and high practical applications. It is used in the treatment of serious infections, e.g., sepsis, infections of the respiratory and urinary tract [36].

The complex of DOPA–melanin with netilmicin was prepared in the following manner [6]: 40 mg of melanin was incubated with 40 ml of drug solutions in 0.067 M phosphate buffer at pH 7.0. The initial concentration of netilmicin was 1 × 10^−3^ M. The control sample contained 40 mg of melanin and 40 ml of buffer without netilmicin. The samples were incubated for 24 h at room temperature. After incubation the suspension was filtered. The amount of netilmicin in filtrate with respect to the control sample was determined. The spectrochemical analysis of the amount of netilmicin in filtrate was performed on an UV–vis spectrophotometer JASCO model V-530. The amount of netilmicin bound to melanin was calculated as the difference between the initial amount of the antibiotic administered to melanin and the amount of the unbound drug in filtrate after incubation. The amounts of netilmicin bound to melanin in DOPA–melanin–netilmicin, [DOPA–melanin–netilmicin]–Cu(II) and [DOPA–melanin–Cu(II)]–netilmicin complexes were [μmol/mg melanin]: 0.48, 0.41 and 0.29, respectively [6].

DOPA–melanin–Cu(II) complexes were obtained as follows [6]: 100 mg of DOPA–melanin was mixed with 100 ml of the metal ion solution containing 1 × 10^−3^ M Cu(II). The samples were incubated for 24 h at room temperature and then filtered. An atomic absorption AAS3 spectrophotometer (Carl Zeiss, Jena) was used to determine the amount of Cu(II) bound to melanin. The amounts of Cu(II) bound to melanin in DOPA–melanin–Cu(II), [DOPA–melanin–netilmicin]–Cu(II) and [DOPA–melanin–Cu(II)]–netilmicin complexes were [μmol/mg melanin]: 0.43, 0.15 and 0.36, respectively [6].

Mixed [DOPA–melanin–netilmicin]–Cu(II) and [DOPA–melanin–Cu(II)]–netilmicin complexes were obtained as follows [6]: DOPA–melanin samples were incubated with the first complexing agent solution for 24 h and then filtered. The obtained complex was incubated for the next 24 h with the second complexing agent solution and filtered after incubation. Based on the amounts of unbound drug and metal ions, the amounts of netilmicin and Cu(II) ions bound to melanin were calculated.

### EPR Measurements

EPR spectra were measured as the first derivatives of absorption on an X-band (9.3 GHz) EPR spectrometer produced by BRUKER Firm. EPR spectra were measured with the microwave power from 0.3 to 200 mW at temperatures in the range of 105–300 K. Changes of amplitudes and linewidths with the microwave power were determined. The continuous microwave saturation of the EPR spectra was done to determine spin–lattice relaxation processes in the samples. The time of spin–lattice relaxation processes decreases with increasing microwave saturation of the EPR line [38].

The total microwave power (*M*_o_) produced by a klystron was 200 mW. The microwave power was changed using different attenuations. The microwave power (*M*) used during the measurement of the individual EPR spectrum was determined from the following formula [38, 39]:

$$ At \, \left[ {{\text{d}}B} \right] = lg \, \left( {M_{\text{o}} /M} \right) $$

The *g*-factor value was determined from the resonance condition as [38, 39]:

$$ g = h{{\nu /\mu }}_{\text{B}} B_{\text{r}} , $$

where *h* is the Planck constant, ν is the microwave frequency, μ_B_ is the Bohr magneton and B_r_ is the resonance magnetic field.

The concentration of the paramagnetic centers (*N*) was determined from the following formula:

$$ N = N_{\text{u}} \left[ {\left( {W_{\text{u}} A_{\text{u}} } \right) /I_{\text{u}} } \right]\left[ {I /\left( {WAm} \right)} \right] , $$

where N_u_ is the amount of paramagnetic centers in the reference—where ultramarine, *W* and *W*_*u*_ are the receiver gains for the melanin sample and the ultramarine, *A* and *A*_*u*_ are the amplitudes of a ruby signal for the sample and ultramarine, *I* and *I*_*u*_ are the integral intensities for the sample and ultramarine and *m* is the mass of the sample [38]. The integral intensity (*I*) of the EPR spectra was calculated by the double integration of the first derivative curves [39]. Ultramarine was used as the reference of free radical concentrations. A ruby crystal as the secondary reference was also used. The ruby crystal was permanently placed in the resonance cavity. For each of the tested samples and ultramarine in the cavity the EPR lines of the ruby crystal were measured.

## Results

**Fig. 1:**
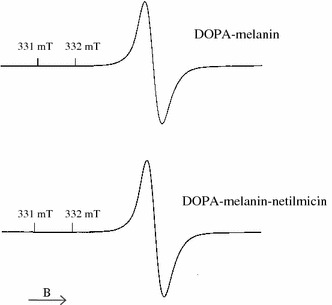
EPR spectra of DOPA–melanin and DOPA–melanin–netilmicin complexes. The spectra were measured with the microwave power of 0.3 mW at the temperature of 300 K

**Fig. 2:**
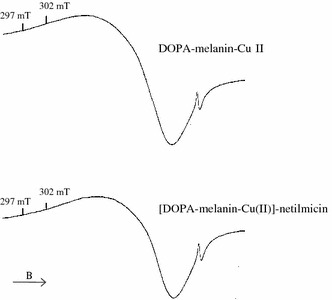
EPR spectra of DOPA–melanin–Cu(II) and [DOPA–melanin–Cu(II)]–netilmicin complexes. The spectra were measured with the microwave power of 0.3 mW at the temperature of 300 K

For DOPA–melanin and DOPA–melanin–netilmicin complexes single asymmetrical EPR lines were measured. EPR spectra of DOPA–melanin and DOPA–melanin complexes with netilmicin are presented in Fig. 1. EPR spectra of DOPA–melanin–Cu(II), [DOPA–melanin–netilmicin]–Cu(II) and [DOPA–melanin–Cu(II)]–netilmicin complexes were superposition of EPR lines of melanin free radicals and copper ions. EPR spectra of DOPA–melanin–Cu(II) and [DOPA–melanin–Cu(II)]–netilmicin complexes are shown in Fig. 2.

**Fig. 3:**
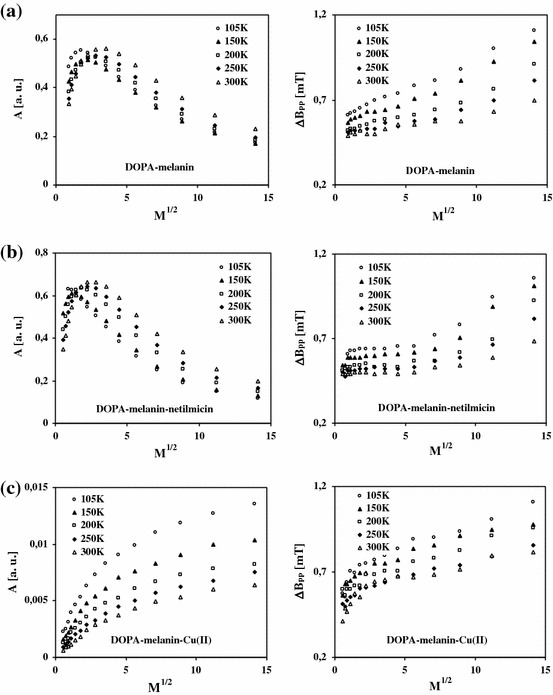
Effect of the microwave power (*M*) on amplitudes (*A*) and linewidths (Δ*B*_pp_) of EPR spectra of DOPA–melanin (**a**), DOPA–melanin–netilmicin (**b**) and DOPA–melanin–Cu(II) (**c**) complexes at temperatures in the range of 105–300 K

**Fig. 4:**
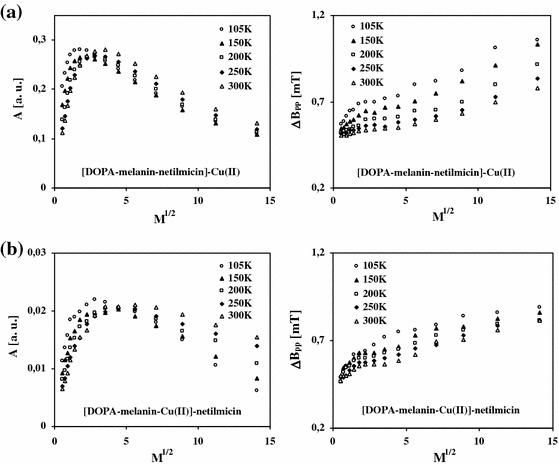
Effect of the microwave power (*M*) on amplitudes (*A*) and linewidths (Δ*B*_pp_) of EPR spectra of [DOPA–melanin–netilmicin]–Cu(II) (**a**) and [DOPA–melanin–Cu(II)]–netilmicin (**b**) complexes at temperatures in the range of 105–300 K

The microwave saturation of EPR spectra of free radicals was tested. EPR spectra of the studied samples change with increasing microwave power. Effects of the microwave power (*M*) on amplitudes (*A*) of EPR lines of DOPA–melanin, DOPA–melanin–netilmicin and DOPA–melanin–Cu(II) complexes at different temperatures in the range of 105–300 K are compared in Fig. 3. Changes of linewidths (Δ*B*_pp_) of EPR spectra of free radicals in DOPA–melanin, DOPA–melanin–netilmicin and DOPA–melanin–Cu(II) complexes with the microwave power at the temperatures (105–300 K) are also presented in Fig. 3. The effect of the microwave power (*M*) on amplitudes (*A*) and linewidths (Δ*B*_pp_) of EPR spectra of DOPA–melanin–netilmicin]–Cu(II) and [DOPA–melanin–Cu(II)]–netilmicin complexes are shown in Fig. 4, respectively. In Fig. 4 data for the measurements at temperatures 105–300 K are compared.

EPR lines of DOPA–melanin–Cu(II) complexes are not saturated (Fig. 3c). For the others samples EPR lines of melanin free radicals are saturated at low microwave powers (Figs. 3a, b, 4). Linewidths of the all measured EPR spectra increase with increasing microwave power at the studied temperatures (Figs. 3, 4).

## Discussion

Stable paramagnetism characterizes DOPA–melanin, DOPA–melanin–netilmicin, DOPA–melanin–Cu(II), [DOPA–melanin–netilmicin]–Cu(II) and [DOPA–melanin–Cu(II)]–netilmicin complexes studied in this work. EPR spectra were measured for these samples at all the tested temperatures. The *g*-factor values were in the range 2.0039–2.0040. Amplitudes (*A*) of EPR lines of melanin paramagnetic centers at 105–300 K decrease in the following order (Figs. 3, 4):

$${\text{DOPA}} {-} {\text{melanin}} {-} {\text{netilmicin}} >{\text{DOPA}} {-} {\text{melanin}}>[ {\text{DOPA}} {-} \hbox{melanin} {-} \hbox{netilmicin}] {-} {\text{Cu}}( {\text{II}} ) >[ {\text{DOPA}} {-} \hbox{melanin}{-}\hbox{Cu}( {\text{II}} )]{-} {\text{netilmicin}} >\,\hbox{DOPA} {-} \hbox{melanin} {-} \hbox{Cu}( {\text{II}} ) .$$

The above correlation confirmed our earlier results for DOPA–melanin complexes with netilmicin and copper(II) ions [6]. Concentrations of paramagnetic centers in DOPA–melanin–netilmicin, DOPA–melanin, [DOPA–melanin–netilmicin]–Cu(II), [DOPA–melanin–Cu(II)]–netilmicin and DOPA–melanin–Cu(II) complexes are [×10^19^ spin/*g*]: 1.8, 1.6, 0.9, 0.1 and 0.1, respectively [6]. EPR measurements at room temperature [6] indicated that the concentration of paramagnetic centers in these samples decreases similarly to the amplitude of the resonance absorption curves at 105–300 K. Netilmicin increases the amplitude and paramagnetic concentration in DOPA–melanin. Paramagnetic Cu(II) ions decrease the amplitude and paramagnetic concentration in both DOPA–melanin and its complexes with netilmicin. This study confirmed (Figs. 3c, 4) the effect of quenching of EPR spectra by Cu(II) described at literature [1, 6, 26]. Similar correlations for amplitudes of EPR lines measured at low temperatures were observed for DOPA–melanin and DOPA–melanin complexes with kanamycin and Cu(II) [11]. The effect of netilmicin and kanamycin [11] on the amplitude of EPR lines indicates the activity of free radicals during complexing of melanin with drugs. The formation of DOPA–melanin complexes with netilmicin and kanamycin [11] is modified by copper(II) ions. It is expected that melanin paramagnetic centers are responsible for toxic effects in human organism. Netilmicin and kanamycin [11] increase the toxic effects during therapy, because of the highest concentration in DOPA–melanin–antibiotic complexes.

The observation of changes of parameters of EPR lines with the microwave power gives us information about type of broadening (inhomogeneous or homogeneous) [38]. The amplitude of inhomogeneously broadened EPR lines increases with increasing of microwave power and after reaching the maximum does not change [38]. The linewidth of the inhomogeneously broadened EPR line does not depend on the microwave power. Isolated spin packets exist in the samples with inhomogeneous broadened EPR spectra [39]. Such spin packets were not found in DOPA–melanin complexes with netilmicin and kanamycin [11] and Cu(II). The amplitude of homogeneously broadened EPR lines increases with increasing microwave power and after reaching the maximum its value decreases [38]. The linewidth of the homogeneously broadened EPR spectrum increases with increasing microwave power [38]. The decrease in amplitudes of EPR lines at higher microwave powers (Figs. 3a, b, 4) and broadening of EPR lines with increasing microwave power (Figs. 3, 4) indicate that these lines are homogeneously broadened [38]. Similar correlations were obtained for DOPA–melanin complexes with Cu(II) and netilmicin [6] and kanamycin [11] complexes at room temperature.

EPR lines of samples with different spin–lattice relaxation times differ in microwave saturation [38]. The spin–lattice relaxation time decreases with increasing of microwave power of the saturation of EPR spectrum [38]. The absence of the microwave saturation of EPR lines of DOPA–melanin–Cu(II) complexes at the used microwave power (Fig. 3c) indicates that the fast spin–lattice relaxation exists in this sample. Fast interactions of unpaired electrons with the diamagnetic molecular lattice are characteristic of DOPA–melanin–Cu(II) complexes. It was found that slow relaxing paramagnetic systems exist in the organic matter of DOPA–melanin (Fig. 3a), DOPA–melanin–netilmicin (Fig. 3b) and DOPA–melanin complexes with both netilmicin and Cu(II) (Fig. 4). The lower spin–lattice relaxation times in melanin structure appear at higher temperatures, because the lines saturate at higher microwave powers (Figs. 3a, b, 4). Netilmicin did not considerably change spin–lattice relaxation processes in DOPA–melanin complexes, because the correlation between amplitudes of EPR spectra and the microwave power was similar for DOPA–melanin and its complexes with netilmicin (Figs. 3a, b, 4).

The performed EPR examination of model DOPA–melanin complexes with netilmicin and Cu(II) ions expanded our knowledge about the paramagnetic properties of these polymer samples. It is expected that eumelanin naturally existing in human organism reveals a similar behavior. It can be concluded that paramagnetic center systems in eumelanin biopolymer reveal complex character and consist of *o*-semiquinone free radicals and biradicals. EPR studies at room temperature indicated that paramagnetic centers of eumelanin play an important role during the formation of the complex between melanin and netilmicin [6]. The amount of paramagnetic centers in melanin complexes with netilmicin is higher than that in the original DOPA–melanin. Our present study performed at low temperatures confirmed this effect. Amplitudes (*A*) and integral intensities (*I*) of EPR lines of DOPA–melanin–netilmicin complexes are higher than amplitudes and integral intensities of EPR lines of model eumelanin—DOPA–melanin. The increase in the paramagnetic center concentration in melanin after complexing by netilmicin is the negative effect, which may be responsible for toxic free radical reactions in tissues. Our earlier [6] and present spectroscopic studies of melanin samples at room and low temperatures, respectively, showed that copper(II) ions decrease the amount of paramagnetic centers in DOPA–melanin complexes with netilmicin. Results similar to the aforementioned behavior at room and low temperatures were obtained for DOPA–melanin complexes with kanamycin [11]. Spin–lattice relaxation processes in DOPA–melanin complexes with netilmicin and kanamycin [11] and Cu(II) are similar. Slow spin–lattice relaxation processes exist in the organic matter of DOPA–melanin complexes with these two drugs and Cu(II), while fast spin–lattice relaxation processes characterize the metal ion systems in these polymer samples. The acceleration of spin–lattice relaxation processes in the organic part of DOPA–melanin complexes with netilmicin and kanamycin [11] appears at higher temperatures. The continuous microwave saturation of EPR spectra indicated that slow spin–lattice relaxation processes also characterize DOPA–melanin complexes with gentamicin [7] and dihydrostreptomycin [10].

The performed studies bring to light the usefulness of EPR spectroscopy in pharmacy. It was shown that this physical experimental method can be widely applied in examining melanin complexes with drugs. Free radical concentrations, their distributions in the polymer and their magnetic interactions may be tested by the use of an EPR spectrometer. EPR spectroscopy may be a useful technique to examine melanins linked with drugs. The obtained results indicate progress in the knowledge and understanding of melanin–drug interactions.

## Conclusions

The X-band (9.3 GHz) EPR studies melanin paramagnetic centers of DOPA–melanin and its complexes with netilmicin and Cu(II) indicated out that

1. Fast spin–lattice relaxation processes exist in DOPA–melanin–Cu(II) complexes.
2. Slow spin–lattice relaxation processes exist in DOPA–melanin, DOPA–melanin–netilmicin, [DOPA–melanin–netilmicin]–Cu(II) and [DOPA–melanin–Cu(II)]–netilmicin complexes.
3. EPR lines of DOPA–melanin and the tested melanin complexes with Cu(II) and netilmicin are homogeneously broadened.
4. The acceleration of spin–lattice relaxation processes occurs with increasing of temperature in the range of 105–300 K.
5. EPR spectroscopy is a useful technique to examine melanin complexes with drugs.
